# Clinical Significance of TUBGCP4 Expression in Hepatocellular Carcinoma

**DOI:** 10.1155/2022/9307468

**Published:** 2022-12-08

**Authors:** Chuanjun Zheng, Jiaxi Zhang, Fusheng Jiang, Di Li, Caimei Huang, Xuefeng Guo, Xiaonian Zhu, Shengkui Tan

**Affiliations:** ^1^Department of Epidemiology and Health Statistics, School of Public Health, Guilin Medical University, Guilin, 541199 Guangxi, China; ^2^Guilin Center for Disease Control and Prevention, Guilin, 541001 Guangxi, China; ^3^Department of Epidemiology and Health Statistics, School of Public Health, Central South University, Changsha 410005, China

## Abstract

We aim to investigate the expression and clinical significance of the tubulin gamma complex-associated protein 4 (TUBGCP4) in hepatocellular carcinoma (HCC). The mRNA expression of TUBGCP4 in HCC tissues was analyzed using The Cancer Genome Atlas (TCGA) database. Paired HCC and adjacent nontumor tissues were obtained from HCC patients to measure the protein expression of TUBGCP4 by immunohistochemistry (IHC) and to analyze the relationship between TUBGCP4 protein expression and the clinicopathological characteristics and the prognosis of HCC patients. We found that TUBGCP4 mRNA expression was upregulated in HCC tissues from TCGA database. IHC analysis showed that TUBGCP4 was positively expressed in 61.25% (49/80) of HCC tissues and 77.5% (62/80) of adjacent nontumor tissues. The Chi-square analysis indicated that the positive rate of TUBGCP4 expression between HCC tissues and the adjacent nontumor tissues was statistically different (*P* < 0.05). Furthermore, we found that TUBGCP4 protein expression was correlated with carbohydrate antigen (CA-199) levels of HCC patients (*P* < 0.05). Further, survival analysis showed that the overall survival time and tumor-free survival time in the TUBGCP4 positive group were significantly higher than those of the negative group (*P* < 0.05), indicating that the positive expression of TUBGCP4 was related to a better prognosis of HCC patients. COX model showed that TUBGCP4 was an independent prognostic factor for HCC patients. Our study indicates that TUBGCP4 protein expression is downregulated in HCC tissues and has a relationship with the prognosis of HCC patients.

## 1. Introduction

Hepatocellular carcinoma (HCC) is one of the most common and deadly digestive tract tumors, accounting for 90% of primary liver cancer, and is the fourth leading cause of cancer-related death worldwide [1–3]. The low rate of early diagnosis is a major factor leading to a poor overall survival of HCC patients, which limits the effectiveness of treatment [4]. Therefore, we need to have a better understanding of HCC to improve the early diagnosis and treatment of HCC. At present, the complex genome map of HCC has been widely studied to find the correlation between genes and the prognosis of patients [5]. It is of great theoretical significance and clinical application value to search for predictive markers for recurrence and metastasis of HCC and explore potential targets of intervention therapy for improving the prognosis of patients with HCC [6–9]. The pathogenesis, prognostic markers, molecular targets, and carcinogenesis mechanism of HCC have been extensively studied [10–13], but the exact molecular mechanism of HCC is still not fully understood.

Tubulin gamma complex-associated protein 4 (TUBGCP4) is a part of the *γ*-tubulin ring complex, which is essential for the microtubule nucleation of the centrosome. In particular, it plays a crucial role in the formation of cilium structure, so the assembly or function defects of cilia may cause syndromic cilia lesions [14]. Under normal physiological conditions, TUBGCP4 functions in embryonic development and retinal dynamic balance and has a gene dosage effect [15–17]. The abnormal expression of TUBGCP4 can lead to many diseases, especially malignant tumors. It has been found that TUBGCP4 protein is abnormally expressed in ovarian epithelial carcinoma [18], mantle cell lymphoma, and myeloma [19] and is closely related to tumor cell growth and metastasis. However, there are few studies on TUBGCP4 in HCC.

In this study, TUBGCP4 expression was detected in HCC and adjacent nontumor tissues by bioinformatics and immunohistochemistry (IHC) methods. We aimed to analyze the relationship between TUBGCP4 and clinicopathological characteristics as well as HCC prognosis so as to provide a theoretical basis for the clinical application of TUBGCP4 in HCC diagnosis.

## 2. Materials and Methods

### 2.1. Tissue Samples

Eighty paired HCC and adjacent nontumor tissues were collected from HCC patients that had not received any type of treatment before surgery in the First Affiliated Hospital of Guilin Medical University. All patients had complete clinical data that are shown in Table 1 and signed the written informed consent. This study was approved by the Ethics Committee of Guilin Medical University (GLMC2014003) in accordance with the Declaration of Helsinki in 1975.

### 2.2. Immunohistochemistry (IHC)

TUBGCP4 protein was detected by IHC method according to the previous study [20]. To assess the expression of TUBGCP4 in tissues, five different visual fields of view were randomly selected at 400× under microscopy for analysis. The total number of cells and the number of positive cells were counted to score according to the percentage of positive cells. The scores for the percentage of positively stained cells were 0 for ≤5%, 1 for 6-25%, 2 for 26-50%, 3 for 51-75%, and 4 for >75%, respectively. According to the intensity of staining, the scores of unstained, light brown, medium brown, and dark brown were 0, 1, 2, and 3, respectively. Finally, the products of the percentage of positively stained cells and staining intensity scores were used as the final TUBGCP4 staining score: 0 was (-), 1-4 was (+), 5-8 was (++), and 9-12 was (+++). The positive and negative expressions of TUBGCP4 were defined as scores >4 and ≤4, respectively.

### 2.3. Bioinformatic Analysis

The HCC gene expression profile files were downloaded from The Cancer Genome Atlas (TCGA) database, including 374 samples of HCC and 50 samples of normal liver tissues, and then the data were collated by R language package, and the differences were analyzed. We downloaded RNA sequence data of HCC patients and analyzed them with Gene Set Enrichment Analysis (GSEA) v4.1.0 software. The significant genes were defined as *P* < 0.05 and FDR < 0.25. At the same time, to understand the potential function of TUBGCP4, Metascape was used for Gene Ontology (GO) of target genes. Subsequently, we explored the proteins that interact with TUBGCP4 on https://string-db.org/cgi/input.pl and visualized them in the PPI network by Cytoscape v3.7.2 software.

### 2.4. Statistical Analysis

All the data were statistically processed by SPSS19.0, and the expression difference between HCC and adjacent nontumor tissues was compared by the Chi-square test. The survival probability was estimated by the Kaplan-Meier method, and the survival curve between different groups was analyzed by the Log-rank test. The independent prognostic factors were analyzed by COX proportional hazard model. *P* < 0.05 was defined as statistically significant.

## 3. Results

### 3.1. Expression of TUBGCP4 in HCC Tissues

**Figure 1:**
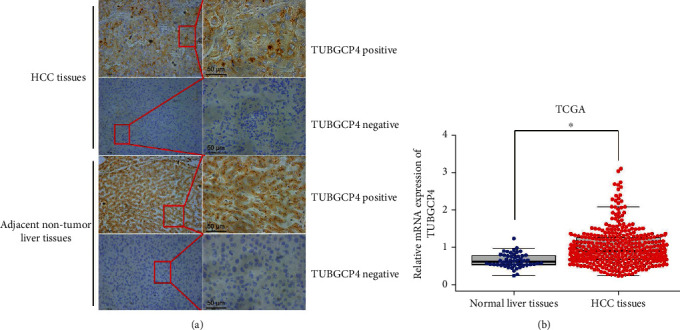
TUBGCP4 expression in HCC tissues. (a) Representative TUBGCP4 protein expression was detected in HCC and its adjacent tissues. (b) The expression of TUBGCP4 in HCC and normal liver tissues from TCGA database, ^∗^ indicates *P* < 0.05.

We first detected the expression of TUBGCP4 in 80 paired HCC and adjacent nontumor tissues by IHC assay. The positive expression of TUBGCP4 in HCC and adjacent nontumor tissues was 61.25% (49/80) and 77.5% (62/80), respectively. TUBGCP4 protein was mainly located in the cytoplasm (Figure 1(a)). As shown in Table 2, TUBGCP4 protein had a higher expression in the adjacent nontumor tissues than HCC tissues (*χ*^2^ = 7.475, *P* = 0.006). We then confirmed the mRNA expression of TUBGCP4 in HCC and normal liver tissues from the TCGA database and found that TUBGCP4 mRNA expression in HCC tissues was significantly higher than that in normal liver tissues (*P* < 0.05, Figure 1(b)).

### 3.2. Relationship between TUBGCP4 Expression and Clinicopathological Characteristics of HCC Patients

We found that TUBGCP4 expression was not related to age, gender, recurrence, vascular tumor thrombus, HBsAg, portal vein tumor thrombus, liver cirrhosis, tumor capsule, distant metastasis, tumor number, pathological grade, TNM stage, carcinoembryonic antigen (CEA), or alpha-fetoprotein (AFP) levels in HCC patients but correlated with carbohydrate antigen (CA-199) levels (*P* < 0.05). Patients with CA − 199 ≤ 37 U/mL had a higher positive expression of TUBGCP4 (Table 1).

### 3.3. Relationship between TUBGCP4 Expression and the Prognosis of HCC Patients

**Figure 2:**
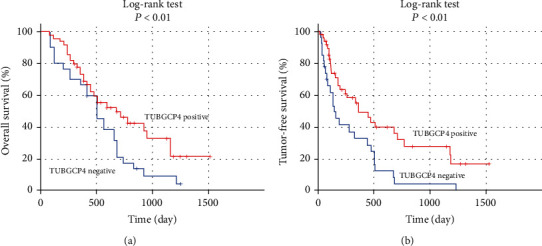
Association between TUBGCP4 expression and the prognosis of HCC patients. (a) Kaplan-Meier model was used to analyze the effect of TUBGCP4 expression on the overall survival of HCC patients. (b) Kaplan-Meier model was used to analyze the effect of TUBGCP4 expression on tumor-free survival of HCC patients.

To investigate the prognostic value of TUBGCP4 in HCC, we performed a Kaplan-Meier model to analyze the effect of TUBGCP4 expression on the prognosis of HCC patients based on IHC results. Postoperative overall survival analysis found that the median survival time of patients in the TUBGCP4 negative group was 506 days (95% CI: 379.794-632.206), and that of patients in the TUBGCP4 positive group was 684 days (95% CI: 365.625-1002.375). Further, Log-rank test showed that the overall survival rate of HCC patients in the TUBGCP4 positive group was significantly higher than that in the negative group (*P* = 0.032, Figure 2(a)). We also found that the postoperative tumor-free survival rate of HCC patients in the TUBGCP4 positive group was significantly higher than that in the negative group (*P* = 0.003, Figure 2(b)).

In addition, we conducted a COX proportional hazard analysis and found that TUBGCP4 was an independent prognostic factor for HCC patients (*P* = 0.040), with an OR value of 0.551 (95% CI: 0.312-0.973, Table 3).

### 3.4. The Main Enrichment Pathways of TUBGCP4 in HCC Tissues

**Figure 3:**
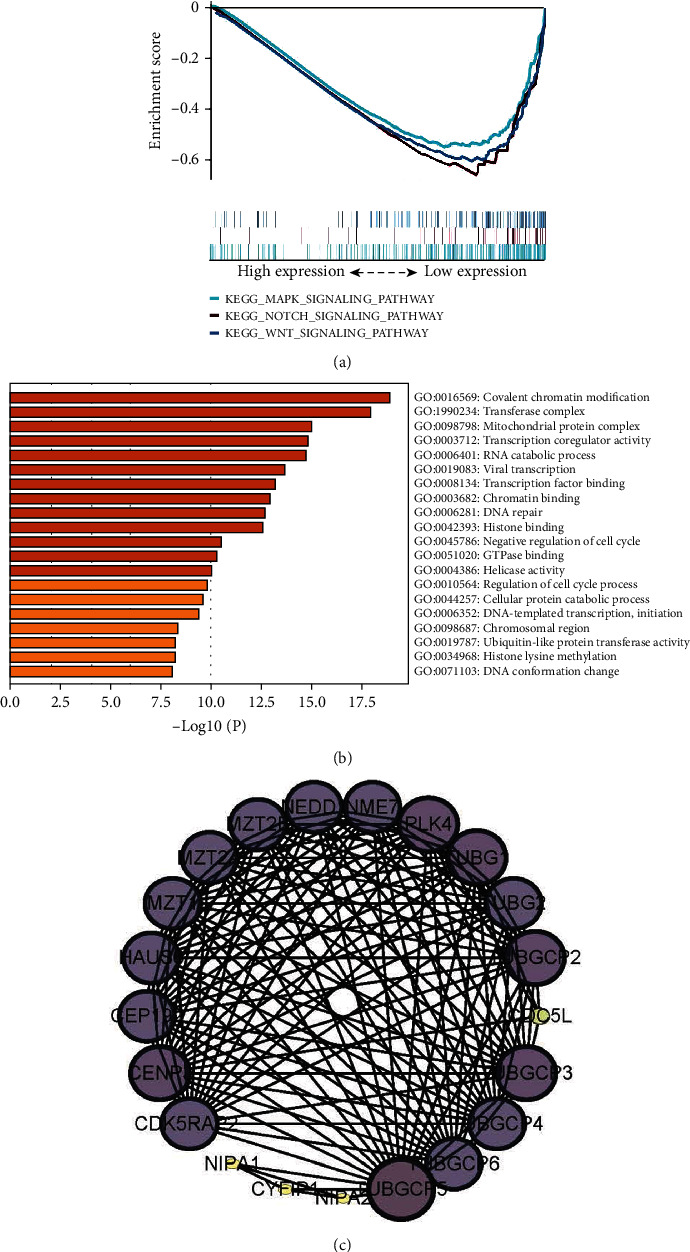
Bioinformatic analysis of TUBGCP4 in HCC. (a) The main enriched GSEA pathways. (b) GO analysis. The darker the color, the closer the target gene is involved. (c) Protein-protein interaction (PPI) network.

In order to clarify the biological functions of TUBGCP4, the effect of TUBGCP4 on the KEGG pathway was analyzed by GSEA. According to the GSEA consequences of TUBGCP4, 44 significantly downregulated enrichment pathways were screened, including “NOTCH signaling pathway,” “WNT signaling pathway,” and “MAPK signaling pathway” (Figure 3(a)). Since, they are the three important tumor-related pathways, which supports that TUBGCP4 might be involved in the occurrence and development of HCC. In addition, GO analysis showed that TUBGCP4 was enriched in biological processes of HCC, such as “covalent chromatin modification,” “transferase complex,” and “mitochondrial protein complex” (Figure 3(b)).

We also constructed a possible protein-protein interaction (PPI) network to study the mechanism of TUBGCP4 in HCC and concluded that TUBGCP4 was closely related to the expression of nonmetastatic cells 7 (NME7), polo-like kinase 4 (PLK4), and other proteins in HCC (Figure 3(c)).

## 4. Discussion

Genetic instability is a common feature of malignant tumors, and centrosome abnormalities are related to the induction of aneuploidy in various human malignant tumors [19, 21, 22]. In structural centrosome proteins, TUBGCP4 encodes *γ*-tubulin complex-associated protein 4, which is a template for closely related *α* and *β* tubulin microtubule polymerization, and its expression is associated with centrosome aberrations [23]. In recent years, some studies have shown that TUBGCP4 is overexpressed in various malignant tumors and considered to be an oncogene. But the expression of TUBGCP4 might be decreased due to abnormal (exclude exon 16) and unstable mRNA production. However, the production of normal mRNA containing exon 16 is not entirely nonexistent and is also produced by the same allele. According to the American Society of Medical Genetics and Genomics [24], this meaningless mutation can be classified as a possible pathogenic mutation because it is a predicted zero mutation in the TUBGCP4 gene, in which loss of function is a known mechanism of microcephaly and chorioretinopathy type 3 (MCCRP3) [25]. Therefore, as a component of the *γ*-tubulin ring complex, the analysis of the expression of TUBGCP4 may be helpful to analyze the centrosome aberration of malignant tumors [26], and they are of great significance to the prognosis of various malignant tumors.

Chromosomal number changes occur in almost all types of human malignancy, including the acquisition and loss of entire chromosomes [22]. Therefore, the regulation mechanism of fidelity of mitotic chromosome separation in mammalian cells is a hot topic in tumor biology research. Importantly, Kramer et al. found that the abnormality of the centrosome is related to the occurrence of aneuploidy in different solid tumors caused by poor chromosome segregation in the anaphase of mitosis [21]. A previous study has reported that TUBGCP4 mutations existed in epithelial ovarian cancer, and TUBGCP4 involved in tubulin processing was essential for cell growth [18]. In the study of mantle cell lymphoma, TUBGCP4 expression was found to be associated with both tetraploidization and high levels of centrosome aberrations [19]. For patients with negative 18F-FDGPET/CT tumor localization but positive TUBGCP4 expression, the detection of tumor markers may be helpful for the localization of tumor origin [27]. These studies suggested that the abnormal expression of the TUBGCP4 gene may lead to abnormal centrosome, which may induce aneuploidy of human malignant tumors and then lead to malignant transformation of cells [26, 28].

In this study, we found that the mRNA expression of TUBGCP4 in HCC tissues was higher than that in normal liver tissues by bioinformatic analysis, while IHC results showed that the protein expression of TUBGCP4 in HCC tissues was lower than that in adjacent nontumor tissues. Such a result may be due to the different expression of TUBGCP4 caused by posttranslational modification, or it may be due to the small sample size in our study. When we analyzed the relationship between TUBGCP4 expression and the clinicopathological characteristics of HCC patients, we found that TUBGCP4 expression was correlated with CA-199 levels. CA-199, a carbohydrate antigen secreted by digestive tract tumor cell lines, is very low in normal adults (<37 U/mL). When malignant lesions occur in the body, the content of CA-199 in tumor tissues is greatly increased. So its content has become one of the main specific indicators for clinical diagnosis of HCC [29]. Our results imply that TUBGCP4 can be used as a diagnostic marker of HCC through the interaction with CA-199.

GSEA results showed that TUBGCP4 regulated hepatocytes through the MAPK, NOTCH, and WNT signaling pathway. They are the three key cascades regulating cancer progression and have been reported to be important in metastasis of many tumors including HCC, non-small-cell lung cancer and colorectal cancer [30–34]. Further, GO analysis indicated that TUBGCP4 was enriched in biological processes of HCC, such as “covalent chromatin modification,” “transferase complex,” and “mitochondrial protein complex”. These regulatory processes are regulated by a variety of enzymatic proteins whose mutation or abnormal expression can easily lead to a variety of diseases [35], including HCC. Subsequently, to further explore the mechanism of TUBGCP4, we constructed a hypothetical protein network and found that TUBGCP4 plays a role in promoting cancer cell growth and proliferation.

Moreover, we found that the total postoperative survival time and tumor-free survival time in the TUBGCP4 positive group were significantly longer than that of the TUBGCP4 negative group. Further COX analysis showed that TUBGCP4 was an independent prognostic factor for HCC patients. However, whether TUBGCP4 can be used as a potential prognosis marker of HCC still needs to be further verified by prospective, multicenter, and large sample experimental methods.

## 5. Conclusion

The downregulation of TUBGCP4 expression is correlated with the prognosis of HCC patients. TUBGCP4 is expected to become a new molecular marker for predicting the prognosis of HCC patients in the future.

## Figures and Tables

**Table 1:** Association between the expression of TUBGCP4 and clinicopathological features of HCC patients.

Variables	Total	TUBGCP4 staining		*χ*2	*P*
		Positive	Negative		
*Gender*					
Male	75	47	28	0.014	0.905
Female	5	3	2		
*Age (year)*					
<50	48	28	20	0.889	0.346
≥50	32	22	10		
*Recurrence*					
No	27	18	9	0.302	0.583
Yes	53	32	21		
*Vascular tumor thrombus*					
No	21	14	7	0.211	0.646
Yes	59	36	23		
*HBV infection*					
No	13	8	5	0.002	0.968
Yes	66	41	25		
*Tumor number*					
<2	75	47	28	0.014	0.905
≥2	5	3	2		
*Portal vein tumor thrombus*					
Yes	79	49	30	0.608	0.436
No	1	1	0		
*Liver cirrhosis*					
No	42	28	14	0.655	0.418
Yes	38	22	16		
*Tumor capsule*					
No	30	19	11	0.014	0.905
Yes	50	31	19		
*Distant metastasis*					
No	76	48	28	0.281	0.596
Yes	4	2	2		
*Tumor grade*					
I + II	11	7	4	0.007	0.933
III + IV	69	43	26		
*TNM stage*					
T1 + T2	74	47	27	0.432	0.511
T3 + T4	6	3	3		
*CA-199 (U/mL)*					
≤37	62	35	27	4.301	**0.038**
>37	18	15	3		
*CEA (ng/mL)*					
≤5	74	46	28	0.048	0.826
>5	6	4	2		
*AFP (ng/mL)*					
<400	40	26	14	0.213	0.644
≥400	40	24	16		

Notes: Bold values indicate significance.

**Table 2:** TUBGCP4 expression in paired HCC tissues and adjacent nontumor tissues.

HCC tissues	Adjacent normal tissues		Total
	Positive	Negative	
Positive	33	16	49
Negative	29	2	31
Total	62	18	80

Notes: *P* = 0.006. *P* value is based on the McNemar *χ*^2^ tests. HCC: hepatocellular carcinoma.

**Table 3:** COX regression analysis for overall survival of HCC patients after surgery.

Variables	*B*	Wald	*P* value	OR	95% CI	
					Lower	Upper
TUBGCP4	-0.596	4.225	**0.040**	0.551	0.312	0.973

Notes: Bold values indicate significance.

## Data Availability

The datasets used and analyzed during the current study are available from the corresponding authors on reasonable request.
